# Asthma and Chronic Rhinosinusitis: How Similar Are They in Pathogenesis and Treatment Responses?

**DOI:** 10.3390/ijms22073340

**Published:** 2021-03-24

**Authors:** Andrea Matucci, Susanna Bormioli, Francesca Nencini, Fabio Chiccoli, Emanuele Vivarelli, Enrico Maggi, Alessandra Vultaggio

**Affiliations:** 1 Immunoallergology Unit, University Hospital Careggi, 50134 Florence, Italy; susannabormioli@gmail.com (S.B.); francescanencini@hotmail.com (F.N.); emanuele.vivarelli@gmail.com (E.V.); vultaggioalessandra@gmail.com (A.V.); 2 Immunology and Cellular Therapy Unit, University Hospital Careggi, 50134 Florence, Italy; fabio.chiccoli@unifi.it; 3 Immunology Department, Children Hospital Bambino Gesù, IRCCS, 00165 Rome, Italy

**Keywords:** asthma, chronic rhinosinusitis, nasal polyps, type 2 inflammation, biological agents

## Abstract

Severe asthma and rhinosinusitis represent frequent comorbidities, complicating the overall management of the disease. Both asthma and chronic rhinosinusitis (CRS) can be differentiated into endotypes: those with type 2 eosinophilic inflammation and those with a non-type 2 inflammation. A correct definition of phenotype/endotype for these diseases is crucial, taking into account the availability of novel biological therapies. Even though patients suffering from type 2 severe asthma—with or without CRS with nasal polyps—significantly benefit from treatment with biologics, the existence of different levels of patient response has been clearly demonstrated. In fact, in clinical practice, it is a common experience that patients reach a good clinical response for asthma symptoms, but not for CRS. At first glance, a reason for this could be that although asthma and CRS can coexist in the same patient, they can manifest with different degrees of severity; therefore, efficacy may not be equally achieved. Many questions regarding responders and nonresponders, predictors of response, and residual disease after blocking type 2 pathways are still unanswered. In this review, we discuss whether treatment with biological agents is equally effective in controlling both asthma and sinonasal symptoms in patients in which asthma and chronic rhinosinusitis with nasal polyps coexist.

## 1. Introduction

Bronchial asthma (BA) is a chronic airway inflammatory disease characterized by the influx of cells, such as lymphocytes, eosinophils, and mast cells and, in a subgroup of patients, of neutrophils, in the bronchial wall [1,2]. The chronic inflammatory process leads to so-called airway remodeling [3]. Asthma is a variable condition in terms of clinical presentations (phenotypes) and distinct underpin pathophysiological mechanisms (endotypes). In fact, based on the biological mechanisms underlining the disease, asthma can be classified as a type 2 (eosinophilic) or non-type 2 (non-eosinophilic) endotype [4,5]. The endotypes referred to as “type 2 disease” are represented by an allergic variant either with or without eosinophilia and by the eosinophilic endotype without allergy [6,7]. In type 2 asthma endotypes, the biological mechanism involved in the inflammatory process is driven by T helper type 2 (Th2) cells, type 2 innate lymphoid cells (ILC2) and type 2 cytokines, including interleukin (IL)-4, IL-5, IL-9 and IL-13 [8]. Biomarkers, such as absolute eosinophil count in peripheral blood, total and specific IgE, and fractional exhaled nitric oxide (FeNO), may be used as indicators of type 2 asthma endotypes and help predict response to biologic therapies, now available for this variant [9,10].

Comorbidities in severe asthma complicate the overall management of the disease. Among them, chronic rhinosinusitis (CRS) has been reported to be frequent comorbidity, and there are data indicating that the presence of CRS is associated with worse outcomes in patients with asthma, more specifically with an increased risk of frequent exacerbations [11,12,13,14]. Phenotypically, CRS is classified as with or without nasal polyps (CRSwNP or CRSsNP), representing approximately 20% and 80%, respectively, of the disease [15,16]. Similar to asthma, CRS can be further differentiated into endotypes. Those with a type 2 eosinophilic inflammation account for about 80% of patients with nasal polyposis, whereas CRSsNP, often characterized by type 1 or type 3 inflammation, is associated with the presence of neutrophils and is regulated by elevated levels of IL-6, IL-8, IL-17, and tumor necrosis factor (TNF)-α [17,18,19]. In addition, in CRS, as observed in asthma, a remodeling process of sinonasal tissues occurs consisting of goblet cell hyperplasia, epithelial barrier abnormalities, basal membrane thickening and polyp formation [20]. Like asthmatic patients, the majority of CRS patients can obtain disease control with conventional treatment. However, a proportion of patients have poor or no control, even with maximal medical therapy and surgery. Patients with CRSwNP with a classical type 2 endotype are usually much more resistant to current therapies, exhibiting a high recurrence rate and, therefore, are considered to have difficult-to-treat rhinosinusitis [15].

A correct definition of asthma and CRS phenotype/endotype is crucial, taking into account the availability of novel biological therapies, such as anti-IgE, anti-IL-5/IL-5Rα and anti-IL4/IL-13Rα, which are dedicated to patients who do not respond to conventional asthma or CRSwNP therapies [21,22,23,24]. Even though patients suffering from type 2 severe asthma with or without CRSwNP significantly benefit from treatment with biologics in terms of clinical improvement, steroid-sparing effect, etc., the existence of high-responders, responders, and nonresponders to these drugs has been clearly demonstrated [25,26].

In this review, we discuss whether treatment with biological agents is equally effective in controlling both asthma and sinonasal symptoms in patients in which asthma and CRSwNP coexist.

## 2. Pathogenic Mechanisms of Upper and Lower Airway Inflammation in Asthma and CRS

The immunopathogenesis of inflammatory processes behind BA and CRS has been clearly defined and, in the great majority of cases, is characterized by type 2 inflammation [27]. Type 2 inflammation is characterized by the presence of cellular infiltration as the result of a complex network of traditional mediators (prostaglandin-D2 (PGD2), leukotrienes, histamine, etc.), key type 2 cytokines (IL-4, IL-5, and IL-13), and chemokines (CCL-3, CCL-5, CCL-11) [28]. The production of type 2 cytokines is sustained by several cellular actors, such as Th2 (both effector memory Th2 recruited from the blood and resident memory Th2 lymphocytes, ILC2, innate-like lymphocytes (ILL) as well as effector cells, namely represented by mast cells, basophils, and eosinophils [29,30,31,32]. Eosinophilic airway inflammation is the hallmark of disease severity in a subset of individuals with severe asthma, and a direct relationship between eosinophil count and the frequency of asthma exacerbations has been demonstrated [33]. It has been shown that, at least in allergic forms, IgE antibodies influence the functioning of several immune and structural cells of the bronchial wall. IgEs are primarily responsible non only for the acute phase but also for the chronic phase of inflammation characteristic of BA [34]. A role of IgE antibodies has also been proposed for CRSwNP [35].

Recently, much attention has been dedicated to IL-5- and IL-13-producing ILC2 significantly increased in sputum of patients with severe asthma with uncontrolled eosinophilia despite treatment with high-dose oral corticosteroids (OCS) [36,37,38]. Notably, also in CRS, an important source of type 2 cytokinesis represented by ILC2s. In fact, experimental data obtained in humans demonstrated that the number of ILC2s is increased in the nasal mucosa of patients with CRSwNP [39,40].

In asthmatic patients, the chronic inflammatory process at the bronchial level leads to airway remodeling where goblet cell hyperplasia, subepithelial collagen deposition, epithelial damage, airway smooth muscle hyperplasia and increased vascularity are the main features of the consequence of chronic stimulation by factors, such as leukotrienes and PGD2, or cytokines and chemokines as transforming growth factor (TGF)-β, IL-1, IL-6, CCL2, CCL3 [3,41,42]. Similar alterations have also been demonstrated in CRS. Indeed, in patients with CRSwNP, histopathological analyses have highlighted that, in addition to diffuse tissue eosinophilia and eosinophilic aggregates, basement membrane thickening, subepithelial edema and fibrosis are evident [43,44]. Therefore, upper and lower airway remodeling is the direct consequence of ongoing or cyclic inflammation and repair occurring in both asthma and CRS.

Besides the type 2 cytokine milieu, it is important to keep in mind other mechanisms as type 1 and type 3 inflammation, which may promote or modulate remodeling. In asthmatics, inflammation and remodeling can be dissociated, as shown by the observation that inflammation, but not remodeling, resolves after few days from allergen challenge [45]. Moreover, there seems to be no correlation between reticular basement membrane thickening and the duration of asthma or, as observed in pediatric asthmatic patients, with the severity of inflammation [46,47]. Even though inflammation is certainly involved in the induction and amplification of the remodeling process, at least in the lower airway tract of asthmatic patients, a part of this activity is initiated by an intrinsic propensity of epithelial cells to aberrantly react to environmental triggers. Epithelial cells have in fact been shown to secrete cytokines, particularly IL-13, TGF-β [48,49], vascular endothelial growth factor (VEGF), metalloproteinases (MMPs) and osteopontin [50,51,52], which in turn activate and transform the underlying mesenchymal cells into fibroblasts [53]. This epithelium-fibroblast signaling pathway, defined as an epithelial-mesenchymal trophic unit (EMTU), may explain the dissociation between inflammation and airway remodeling events [54].

**Figure 1:**
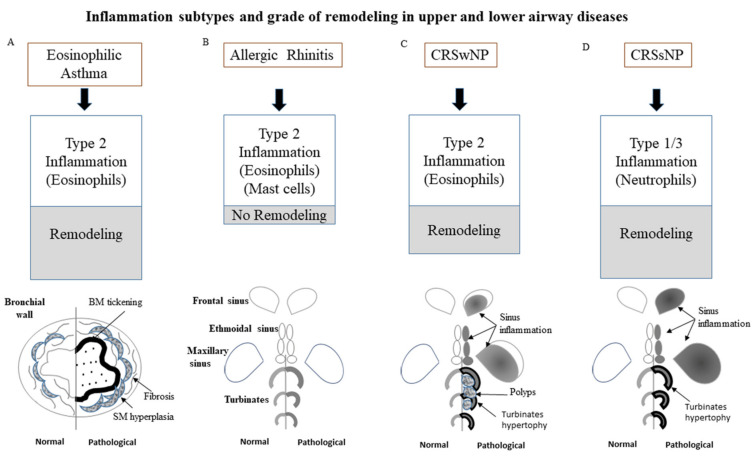
Chronic eosinophilic type 2 inflammatory process at the bronchial wall level of asthmatic patients, both allergic and non, leads to airway remodeling characterized by epithelial damage, goblet cell hyperplasia, subepithelial collagen deposition, airway smooth muscle hyperplasia and increased vascularity (**A**); in allergic rhinitis, despite type 2 inflammation is a disease hallmark, remodeling does not occur (**B**); in CRS type 2 inflammation occurs leading to the development of nasal polyps (CRSwNP), in which the remodeling process is characterized by more evident pseudocyst formation, stromal edema and collagen deposition (**C**); in CRSsNP type 1/3 neutrophilic inflammation is driving force in which remodeling is more evident due to basal membrane thickening, fibrosis and goblet cells hyperplasia (**D**).

If we reconsider the similarity of pathological characteristics between lower and upper respiratory tracts in BA and CRS, some differences emerge between allergic or nonallergic phenotypes and between asthma and rhinitis with or without nasal polyps. As mentioned above, while both allergic asthma and rhinitis are characterized by a type 2 inflammation [55], the remodeling alteration of nasal mucosa does not represent a common feature in allergic rhinitis (AR) even though many actors of remodeling, such as high levels of IL-5 and IL-13 and eosinophil influx, are detectable in nasal fluids [56,57]. In contrast, in CRS with and without nasal polyps, the remodeling process typically occurs, even though more evident in the latter form, due to increased collagen deposition [58,59]. Similar to asthma, the overall aspects of remodeling in CRS as epithelial cell disruption, goblet cell hyperplasia with mucin hypersecretion, basement membrane thickening may be related to disease severity and duration but seem independent from the degree of eosinophilic infiltration [60]. Concerning CRSwNP, the classic type 2 inflammation with eosinophils and IL-4, IL-5, IL-13 overexpression are a hallmark of the disease [61]. On the other hand, the findings that remodeling alterations are also evident in CRS in which neutrophils are the prominent cell type support the view that eosinophils are not essential for the establishment of remodeling [62]. This is in agreement with the evidence that IL-17A, produced by cells of type 3 immune response (Th17, Tc17, ILC3), is the major inducer of IL-8-driven chemotaxis of neutrophils and, in parallel, of fibroblasts’ proliferation and remodeling [63]. In Figure 1, the different types and degrees of inflammation and remodeling are represented for BA and CRS. 

## 3. Biological Agents Targeting Type 2 Inflammation

The differentiation of asthma and CRS into either type 2 or non-type 2 diseases based on the inflammatory pattern has greatly improved the management of these diseases by selecting patients accordingly and taking into account that biologics are only dedicated to the former type. As of date, available approved mAbs are only indicated for type 2 asthma subtypes, and more recently, for eosinophilic CRSwNP [22,23,24]. Such biological agents, approved for the treatment of severe type 2 asthma, include anti-IgE (omalizumab), anti-IL-5 (mepolizumab and reslizumab) anti-IL-5Ra (benralizumab) and anti IL-4/IL-13Ra (dupilumab) [64,65,66,67,68,69,70,71]. Among them, omalizumab selectively binds to free IgE molecules, independently from the specificity, blocking the binding site (Cε3 domain) for FcεRI, modulating and acting upstream of the IgE network and slowing, or preventing, both the early and late allergic inflammatory cascade [23]. The depletion of free IgEs induces downregulation of FcεRI expression not only on mast cells and basophils but also, and more importantly, on both myeloid- and plasmacytoid dendritic cells (DC). Even though indirectly, this reduces the APC activity of these cells, downregulating the allergen-specific T cell response [72,73].

The well-known central role of IL-5 in the differentiation, maturation and survival of eosinophils [74] has laid the groundwork for the development of anti-IL-5 mAbs, such as mepolizumab, reslizumab, and for the anti-IL-5Rα benralizumab. While the effect of the anti-IL-5 mAbs has been related to their ability to indirectly target eosinophils, benralizumab, a humanized fucosylated mAb recognizing the α-subunit of the IL-5 receptor, exerts its effect directly by depleting eosinophils by inducing apoptosis through antibody-dependent cell-mediated cytotoxicity [75]. The efficacy of mAbs targeting IL-5 or its receptor is indisputable in eosinophilic asthma, with more evident clinical results in those patients with a higher percentage of blood and sputum eosinophilia [66]. Although this is true when analyzing large case series of asthmatic patients, the frequency and severity of asthma symptoms in clinical practice may not always be associated with eosinophil count, particularly in patients with blood eosinophilia close to the cutoff point identified as the predicting marker of response.

Taking into account the complex, but the partial interplay between eosinophilic inflammation, remodeling, and the role of the various type 2 cytokines, increasing attention has been dedicated to IL-4 and IL-13, which have been clearly identified as preferential therapeutic targets as they play a central and driving role in the pathogenesis of type 2 inflammation in BA, and CRSwNP [76,77]. In fact, dupilumab, a fully human mAb directed toward the alpha chain of IL-4 receptor used by both IL-4 and IL-13, has been recently introduced for treating type 2 related diseases [22,64]. The clinical effects of dupilumab are related to the fact that these two cytokines play several pathogenic roles: i) IL-4 is an essential factor in the differentiation of Th2 cells; ii) both IL-4 and IL-13 induce the switch towards IgE production; iii) IL-4 and IL-13 induce the expression of adhesion molecules; iv) IL-13 is responsible for smooth muscle hypertrophy and goblet cells hyperplasia.

## 4. Clinical Efficacy of Biological Agents in Asthma and CRS

As previously mentioned, blocking free IgE omalizumab interrupts the IgE-mediated asthma inflammatory cascade at an early stage, thus reducing both early and late asthmatic responses, and improving lung function, asthma control and decreasing the number of exacerbations. A greater effect of exacerbation reduction was observed in patients with high FeNO and periostin levels and high peripheral blood eosinophil counts [78,79,80,81,82]. The clinical use of omalizumab has been recently extended to the treatment of patients with refractory CRSwNP. In fact, in addition to preliminary data of a proof-of-concept study and real-life experience [83,84], two phases 3 studies with omalizumab [23] demonstrated the improvement of −1.08 and −0.89 in nasal polyp score (NPS) and mean daily nasal congestion score (NCS), respectively, with better outcomes and patient-reported assessments of symptom severity. The treatment was also able to improve sinonasal outcome test (SNOT)-22 and overall impact on patients’ quality of life (QoL) [23].

The indirect and direct anti-eosinophilic strategies based on mepolizumab, reslizumab, and benralizumab, are indicated in asthmatic patients exhibiting an eosinophilic phenotype [65,66,67]. Mepolizumab and reslizumab reduce exacerbation rates, improve lung function, reduce OCS exposure, and demonstrate better outcomes in those patients with severe late-onset asthma and CRSwNP [65,85,86,87]. Benralizumab induces rapid depletion of circulating eosinophils and is highly effective in patients with higher exacerbation history, poor lung function, OCS use, CRSwNP, and adult asthma diagnosis [65,66].

Concerning CRSwNP, among mAbs targeting eosinophils, positive results in terms of improvement of nasal symptoms are available for mepolizumab [24], whereas the clinical trial with benralizumab is ongoing.

The last approved mAb, dupilumab, has been demonstrated to significantly reduce the rates of severe asthma exacerbations and OCS use by improving lung function. The greatest treatment benefits have been observed in patients with high peripheral blood eosinophils counts and FeNO levels [88,89]. Of note, dupilumab has been the first biological agent approved for the treatment of CRSwNP. In fact, in adult patients with severe CRSwNP enrolled in the two trials [22], dupilumab reduced polyp size, radiological sinus opacification, and symptom severity. The major mean difference in NPS under dupilumab treatment versus placebo was –2.06, whereas the difference in nasal congestion was −0.89 [22,90]. More important, in the first study, dupilumab also improved the Lund-Mackay computer tomography scores (−7.44) [22].

## 5. Asthma and CRS May Display Different Clinical Outcomes in Response to Biological Treatment

In addition to demonstrating the efficacy of biological agents targeting type 2 inflammation, asthma clinical trials and real-life studies have highlighted a range of responses to treatment [25,26]. The existence of a range of responses is evident when considering the OCS-sparing effect. In fact, a proportion of patients reach complete OCS intake interruption, while others only reduce OCS dose or need to maintain the original OCS dose used before biological treatment [66,85,91,92]. A similar consideration can be made if we analyze the variability of the clinical outcomes in treated subjects. In fact, while some patients experienced exacerbations, some even severe, others remained exacerbation-free throughout treatment. Asthma and nasal symptom responses may also vary between biologicals due to differences in target, dosing, administration interval and patient baseline characteristics, such as body mass index (BMI) and comorbidities. It has been demonstrated that anti-IL-5/IL-5Rα strategies and omalizumab are more likely to be effective in patients with high blood eosinophil count and in those with OCS maintenance dose therapy [66,81,93]. Similar data have been observed for dupilumab also concerning baseline FeNO levels and OCS dose [92,94]. It should be underlined that clinical response may vary over the course of treatment differently between asthma and CRS control.

Many questions regarding responders and nonresponders, predictors of response, and residual disease after blocking type 2 pathways are still unanswered. For example, asthma and CRS can coexist but with different degrees of severity; therefore, efficacy may not be equally achieved. In clinical practice, it is common to experience that patients reach a good clinical response for asthma symptoms, but not for CRS, as reported in some small case series [95]. Moreover, in individual patients, biological mechanisms underpinning asthma and CRS can be only partially similar, not only in terms of severity but also in terms of cellular and molecular “actors” driving the inflammatory process. As mentioned above, the accumulation of eosinophils is a type 2 disease hallmark but not always responsible for the full-blown inflammatory process, including remodeling in different compartments. Directly or indirectly targeting eosinophils can result in partial and/or varying improvement of clinical symptoms [96]. The administered biological dose may represent another potential factor influencing the clinical effects due to a variable capacity of reaching a higher concentration at the tissue level. Indeed, in the clinical trial using 750 mg of mepolizumab intravenously, the need for polyp surgery was significantly reduced, and a significant reduction of endoscopic NPS was observed [97,98]. This hypothesis is supported by Mukherjee et al. [99], who demonstrated an improved response to the weight-based anti-IL-5 mAb reslizumab in patients who still had sputum eosinophilia despite mepolizumab treatment. On the other hand, in a high proportion of EGPA patients treated with mepolizumab 100 mg subcutaneously (sc), a clinically important difference in SNOT22 was observed even though the approved dose in such patients is 300 mg sc [100].

Individual differences in pharmacokinetics and resulting plasma drug levels are additional interfering factors, as demonstrated in other chronic immunomediated diseases in which mAbs are largely used [101,102]. The pharmacokinetics of mAbs is characterized by low extracellular compartment distribution due to their large molecular size and long elimination half-life [102]. The rate and extent of absorption vary between mAbs and between individuals for the same mAb. This raises the possibility that, in some patients with a high BMI, the standard dose may be insufficient to reduce airway and nasal inflammation in the same manner.

**Figure 2:**
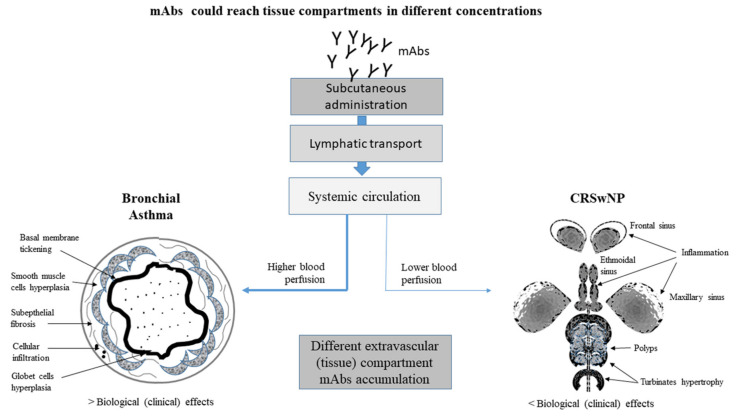
The mAb concentration at bronchial and nasal levels can be influenced by several factors, such as subcutaneous absorption, lymphatic transportation and different blood perfusion in the different organs and tissues, all conditioned by the chronic inflammatory process.

The different effects of mAbs could also be a consequence of a different histopathological substrate at the sinus level compared to that present at the bronchial level. Furthermore, a drug’s variable capacity to effectively reach therapeutic levels in various anatomical districts cannot be ruled out. However, non-negligible differences between asthma and CRS, particularly regarding tissue eosinophil accumulation, tissue remodeling degree and site differences, can condition different clinical outcomes, thus highlighting the need for a tailored therapeutic approach in each patient. In Figure 2, factors influencing tissue diffusion of mAbs are summarized.

One of the key questions is whether the inflammatory process displays similar characteristics at the bronchial and nasal level in patients in which BA and CRS coexist. The upper and lower airway mucosa are structurally similar apart from the presence of airway smooth muscle at the bronchial level. Upper and lower airway biopsy specimens obtained in allergic patients with AR and allergic asthma confirm similar Th2/ILC2 cell-driven inflammation [55]. In agreement, the “allergy march” theory indicates an identical pathogenetic process starting from nasal mucosa in AR and subsequently ending after a variable period of time with the involvement of bronchial airways (BA). The concept of “united airways” was reinforced from the observation that a nasal allergen provocation test induces inflammatory responses not only at the nasal mucosal level but also at the bronchial level, and, conversely, the reciprocal phenomenon occurs at the nasal level after a bronchial allergen provocation test [103,104]. However, this concept does not seem to be automatically applied to all patients in terms of treatment response.

Overall, the data reported in clinical trials summarizing the efficacy of the available biological agents are difficult to compare due to the heterogeneity of the studies’ populations in terms of severity, OCS dose at baseline, etc. Accordingly, we have reported the summary of the effects of biological agents in Table 1.

## 6. Conclusions

In the last few years, the role of the pathogenic mechanisms active in BA and CRS has been further defined, improving the knowledge of potential therapeutic targets. Taking into account the significant proportion of patients in which the two diseases coexist, and the common underpinned cellular and molecular inflammatory network, at least in type 2 forms, it was believed that by using the available biological agents, we could obtain an equivalent therapeutic effect for both asthma and CRS. Although this is true in several patients, in a non-negligible number of them, the improvement of nasal symptoms is less evident, despite reaching satisfactory asthma control. Many questions still need to be answered, specifically referring to the different tissue inflammatory consequences, such as remodeling at the bronchial and nasal levels; the different intensity of the inflammatory process or the existence of two different patterns of inflammation at the bronchial and nasal levels (type 2 and non-type 2 variants); the ability of biological agents to equally reach the different tissue sites. Future studies focusing on tissue samples from the upper and lower airways in response to biological treatment could allow defining the relationship between these two compartments.

## Figures and Tables

**Table 1:** Effects of biologic therapies in asthma and nasal comorbidity.

	Asthma				CRSwNP	Biomarkers		
			
	FEV1	Symp	Exac	OCS Sparing	Symp	Bl Eos	FeNO	IgE
Anti IgE	+	+	+	NA	+	↓	↓↓	↓*
Anti IL-5	+	++	++	+	+	↓↓	↔	↔
Anti IL-5Rα	+	++	++	++	NA	↓↓	↔	↔
Anti IL-4/IL-13	++	++	++	++	++	↑/→	↓↓	↓↓

CRSwNP: chronic rhinosinusitis with nasal polyps; Bl Eos: blood eosinophils; Exac: exacerbations; FEV1: forced expiratory volume in 1 second; NA: not available; OCS: oral corticosteroids; Symp: symptoms; *reduction of free IgE.
